# Analytical Review of Contemporary Fatwas in Resolving Biomedical Issues Over Gender Ambiguity

**DOI:** 10.1007/s10943-018-0616-0

**Published:** 2018-04-21

**Authors:** Taqwa Zabidi

**Affiliations:** School of Theology, Religious Studies and Islamic Studies, Faculty of Humanity and Performing Arts, University of Wales Trinity Saint David, Lampeter Campus, Ceredigion, SA48 7ED UK

**Keywords:** Fatwa, Khuntha, Gender ambiguity, Disorders of Sex Development, Islamic biomedical ethics

## Abstract

Issues of gender ambiguity have been discussed over time from both Islamic and medical perspectives. In Islam, these issues are typically considered in the context of *khunūthah* (literally translated as hermaphroditism). While biomedical studies have appeared to provide a large amount of information on abnormal human biological development, i.e. Disorders of Sex Development (DSDs). However, the connection between these two fields has been given little attention. This research aims to determine the Islamic underpinnings through the fatwa around the globe. Thus, institutional fatwa organisations among Sunni schools of thought at the international, regional and national levels are observed. The fatwas regarding the management of individuals with gender ambiguity, not specifically on DSDs, are chosen and presented accordingly. Based on the findings, the sporadic fatwas from different parts of the world delineate the issue of sex ambiguity and seem to be able to provide general guidelines for management of Muslim patients with DSDs. Three common aspects have been discussed including the methodology of gender assignment, the decision-making process and the surgical and hormonal treatments.

## Introduction

Issues of gender ambiguity have been discussed over time from both Islamic and medical perspectives. In Islam, these issues are typically considered in the context of *khunūthah* (literally translated as hermaphroditism). The classical Islamic delineation is putative in nature. However, current biomedical technology has increased our insight into this complex condition. Biomedical studies have appeared to provide a large amount of information on abnormal human biological development. However, the connection between these two fields has been given little attention. Dynamism of Islamic perspectives is required to resolve biomedical issues over gender ambiguity.

To achieve the objective, this research aims to determine the Islamic underpinnings through the fatwa around the globe. A fatwa is a legal opinion or an Islamic religious decree issued by a *mufti* (religious scholar) on any current issues that have not been explicitly explained in Islamic juridical sources such as the Quran and hadith. Presently, authority of providing of fatwas has been institutionalised at the national, regional and international levels. The discussions entail numerous biomedical issues, but little knowledge has yet to be compiled concerning the Islamic perspectives of the medical conditions related to Disorders of Sex Development (DSDs). Thus, any fatwas from Sunni schools of thought that have been issued regarding the management of individuals with gender ambiguity, not specifically on DSDs, are observed and presented accordingly. Due to the nature of fatwa, most of the answers to the raised questions are responded in a somewhat scattered form, comprising fatwas from across geographical boundaries.

## Gender Ambiguity in Islamic and Biomedical Perspectives

In the medical field, gender ambiguity was formerly known as hermaphroditism. The term hermaphrodite was used in the medical settings to indicate at least three types, namely true hermaphrodites (having both testicular and ovarian tissue with or without ambiguous genitalia), male pseudo-hermaphrodites (having testes with ambiguous genitalia) and female pseudo-hermaphrodites (having ovaries with ambiguous genitalia) (Money 1985). The origin of the terms traces back to the tales of Hermaphroditus, the son of Hermes and Aphrodite in Greek myth (Seymour 2011). The mythical image of the term as monstrous or freakish caused usage avoidance by the affected families (Reis 2007). It also has been a derogatory term such as hybrid, impostor, sexual pervert and unfortunate creature (Reis 2007). These terms are pejorative and controversial to the patients and their families (Aaronson and Aaronson 2010; Hughes 2015).

The term hermaphrodite was gradually less used in medical setting, and a new term, intersex, was coined by Richard Goldsmith in 1917, replacing the previous term (Reis 2007). This new term highlights the ‘discrepancy between the external genitals and the internal genitals (the testes and ovaries)’ (National Institute of the Health 2016). In other words, the Intersex Society of North America (ISNA) (2008) refers to it as ‘a variety of conditions in which a person is born with a reproductive or sexual anatomy that does not seem to fit the typical definitions of female and male’.

However, the definition of intersex itself is restricted, as there is exclusion of anomalous conditions without ambiguous genitalia. Consequently, the European Society for Paediatric Endocrinology and the Lawson Wilkins Paediatric Endocrine Society came to a consensus to replace the term with Disorders of Sex Development (DSDs) (Hughes 2008). This current terminology provides an all-embracing condition of gender ambiguity with or without genital anomalies compared to the previous terms of hermaphrodites and intersex. This is also intended to avoid parental uneasiness with the term intersex putting their children’s identity between male and female (Reis 2007; Hughes 2008). This consensus, also known as the Chicago Consensus, defines DSDs, which is an umbrella term with many different diagnoses, as a set of congenital conditions in which the development of chromosomal, gonadal and anatomical sex is atypical (Hughes 2008).

With the changing of the generic name, nomenclatures related to hermaphroditism have also been replaced with terms referring to chromosomal factors, acknowledging the karyotype analysis in most cases of DSDs (Hughes 2008). Karyotype refers to ‘the chromosome set of an individual or species described in terms of both the number and structure of the chromosomes’ (Concise Medical Dictionary 2010). Many types of sex ambiguity have been categorised under three main diagnostic categories of DSDs, namely 46,XY DSD (previously known as male pseudo-hermaphrodite), 46,XX DSD (previously known as female pseudo-hermaphrodite) and sex chromosome DSD that the sex chromosomes are atypical (Hughes 2008; Lin and Achermann 2009; Achermann et al. 2015).

One in every 4500 births has an abnormality in genital appearance (Öçal 2011). Research shows that a significant number of cases occur in Muslim majority countries. A study in Egypt dating from 1966 to 2009 shows that out of 28,735 patients registered at Medical Genetics Centre, 908 patients of them were clinically confirmed as DSDs cases (Shawky and Nour El-Din 2012). Nasir Al-Jurayyan also reports that DSDs is not uncommon in Saudi Arabia (Al-Jurayyan 2011a, b). In Malaysia, a high frequency of Congenital Adrenal Hyperplasia (CAH) problem—which is one of the most prevalent types of DSDs—is recorded as high as 1:3000 cases compared to the birth rate in Kuala Lumpur (Wu et al. 1994). In Turkey, at least 70 cases with CAH problems were recorded between 1983 and 2002 (Özbey et al. 2004). Over a 5-year period, 156 cases were seen in research conducted at Sudan’s Paediatric Endocrinology Clinic (Abdullah et al. 2012). These figures give a view that Muslims have to deal with the issue of gender ambiguity.

At the first glance, this condition has resemblance characteristics of what is known in Islamic jurisprudence as *khunūthah* (hermaphroditism) or more often than not it is identified as a *khunthā* (a hermaphrodite individual). In Arabic lexicon, *al*-*khunthā* (الخنثى) derives from the word *khanatha* (خنث), which literally means fold and bend something, for example to fold back the mouth of a waterskin (a receptacle used to hold water made of cow or sheep bladder) for drinking (Majmaʿ al-Lughah al-ʿArabiyyah bi al-Qāhirah n.d.). *Khunthā* technically refers to a person who cannot be recognised as male or female and who has both criteria for men and women (Ibn Manẓūr 2010). It is associated with the character of fractured and lack of male features while presenting more than ordinary female characteristics due to retaining dual genitalia (Ibn Mawdūd al-Mūṣilī 1937). Jurists of the four Sunni schools of thought (Ḥanāfī, Mālikī, Shāfiʿī and Ḥanbalī) consistently discussed the condition of *khunthā* in relation to the abnormality of an individual’s sexual anatomy. This condition should not be confused with *muhkhanath* (effeminacy), which is usually paired with suppleness (*tathannīy*) and languidness (*takassur*) in an individual’s way of communication and his/her acts (Ibn Mawdūd al-Mūṣilī 1937; Al-Khin et al. 1992; Rowson 1991).

The scholars regard *khunthā* as an abnormal sexual development rather than acknowledging it as a third gender. The Qurʾānic exegesis explicates that the creation of human is from a single person who is Adam (Qurʾān, 4: 1). From him, Hawwāʾ was created and the procreation continues through biological fertilisation (Qurʾān 75: 37). There are verses mentioning that humans have been created in pairs.

And have We not created you in pairs? (Qurʾān 78: 8).

It continues to explain the ‘pairs’ as the creation of binary gender of human being, i.e. male and female.

O mankind! reverence your Guardian – Lord, who created you from a single person, created, of like nature, His mate, and from them twain scattered (like seeds) countless men and women;- reverence Allah, through whom you demand your mutual (rights), and (reverence) the wombs (that bore you): for Allah ever watches over you (Qurʾān 4: 1).

That He did create in pairs—male and female (Qurʾān 53: 45).

The word *khunūthah* or *khunthā* is never mentioned in the Qurʾān as highlighting the concept of two genders. The scholars unanimously agree that the third gender is simply non-existent and call for the assignation of the appropriate gender for *khunthā* (Ibn Qudamah 1963; Al-Ḥaṭṭāb al-Ruʿīnī 1992; Al-Māwardī 1999; Zakariyya Al-Anṣarī n.d., Ibn ʿAṭiyyah 2001).

The conditions of *khunthā* could be identified in three different forms of genitalia, which are: (1) non-existence of genitalia but an orifice; (2) an ambiguous organ; and (3) two genitalia (Al-Juwaynī 2007; Al-Ḥaṭṭāb al-Ruʿīnī 1992). There are two types of *khunthā,* namely *khunthā wāḍiḥ* (discernible *khunthā*) and *khunthā ghayr wāḍiḥ* or *khunthā mushkil* (intractable *khunthā*). The classifications of *khunthā* depend on the level of difficulty in assigning the gender. *Khunthā wāḍiḥ* is a condition in which the signs of either maleness or femaleness can be identified (Ibn Qudamah 1963). On the other hand, if the signs to demonstrate either one of the genders are obscure, they are identified as *khunthā mushkil.* The term is dedicated to a condition that has no dominant signs, by either urine excretion rules (*ḥukm al*-*mabāl*) or secondary bodily characteristics to overweigh the feebler gender.

The status of *khunthā* raises some questions in several gender-related religious obligations such as in purification, prayer, pilgrimage, modesty, marriage, nursing, transaction, inheritance, criminal law and funeral (Tak 1998). There are nuances of the performance between the two genders in a way that the duties and obligations are not merely rituals, but in themselves are profoundly communal and social activities (Sanders 1991). Therefore, the jurists offered pragmatic solutions for dealing with them, especially for *khunthā mushkil* whose gender is indeterminate (Ibn Qudāmah 1963).

### Issues on DSDs and *Khunūthah*

As history recorded, the legal aspects of *khunūthah* especially in terms of inheritance were discussed even during the pre-Islamic era and later adapted in Islam (Al-Ḥaṭṭāb al-Ruʿīnī 1992; Abū Jaʿfar al-Baghdādī n.d.). Notwithstanding, from the biomedical side, it has not yet been explored comprehensively. Explication of *khunthā* was established by classical jurists predating the present understanding of embryology and biomedical technology (Zainuddin and Mahdy 2016). *Khunthā* emphasises genital ambiguity, including retaining of dual normal genitalia (Ibn Mawdūd al-Mūṣilī 1937). However, DSDs include all types of malformation of sexual development with or without genital ambiguity (Hughes 2008). If DSDs could be associated or defined as *khunūthah*, are those Muslim patients with DSDs with normal genitalia obliged with specific religious obligations for *khunthā mushkil* as long as their sex is undetermined?

Islamic and medical perspectives each have their own guidelines for gender assignment. Ibn Qudāmah (1963), Al-Ḥaṭṭāb al-Ruʿīnī (1992), Al-Māwardī (1999), Al-Sarakhsī (1993) and Al-Suyūṭī (2001) have listed bodily signs that should be taken into consideration in assigning the gender based on the rules on urine excretion and on secondary sexual characteristics. In the medical field, an *optimal gender policy* is widely referred to by the physicians, with current adaptations to accommodate the policy with various background of cases (Money et al. 1955; Meyer-Bahlburg 1998). Adding more to this subtle issue, culture and religion each have great influence on the decision of the most acceptable and practical gender to be accorded to a person with DSDs (Zucker 2002; Shawky and Nour El-Din 2012; Dessouky 2001; Kuhnle and Krahl 2002). This contributes to the delicate aspect of managing patients with DSDs.

The question of who can make the decision to assign gender is vital. It is noted that there exists a gap between the classical understanding of *khunthā* and the accepted current concept of DSDs. In classical Islamic literature, it is widely understood that *khunthā* are those who have dual genitalia or have nothing but an orifice for urination. Conversely, there is very little evidence in medical diagnostic of external dual genitalia cases (Sax August 2002; Ceci et al. 2015) and the non-existence of external genitalia is unknown in the medical literature. Al-Sibāʿi and Al-Barr (1993) claim that we should leave the cases to the experts in the field, i.e. the doctors to deal with them. However, managing Muslim patients with DSDs need not ignore the religious beliefs of patients and parents/guardians. Hence, to what extent should Gatrad and Sheikh’s (2001) suggestion to include Muslim scholars in the medical multidisciplinary team be taken into account? In addition to that, confidentiality in this sense is an important issue that should be given careful attention (Zainuddin and Mahdy 2016).

The most debatable and widely discussed in relation to the contemporary debates of *khunthā* concerns hormonal and surgical treatments. Most treatment is encouraged in Islam, although the Qurʾān has made it clear that alteration of Allah’s creation including sex change is prohibited. This treatment is not altering but correcting physical abnormalities. The concerns here are on the timing of the treatment and its efficacy that need to be addressed clearly (Haneef and Abdul Majid 2015; Mohamed and Mohd Noor 2014a, b). These issues will be analysed thoroughly based on the fatwas provided by various juristic councils from different part of the worlds.

## Methods and Resources

The fatwa is seen as a bridge to close the gap between jurisprudence heritage and the present world. Initially, a fatwa is issued by a jurist consult (*mufti*) who is, (1) a reliable Muslim with high moral values, safe from immorality and dishonour, and (2) has a practical technique for deducing such rulings from the proofs of injunctions (*adillah al*-*aḥkām*) including the Qurʾān, ḥadīth, *ijmāʿ* (consensus) and *qiyās* (analogical reasoning) (Al-Nawawī 1408H). Currently, the position of the jurist consult is beyond a mere individual opinion, as it has become a referral institution for Muslims in resolving contemporary issues (Nanji, Mehmood 2015), in a way that it has been institutionalised regardless the authority of its legal binding.

Hitherto, the idea of institutionalising *ijtihād* is welcomed in every Islamic country, especially in the field of juridical thoughts. The most important element is collective *ijtihād* (*ijtihād jamāʿī*), which is recognised as more reliable than individual *ijtihād* (Hasan 2003). Aznan Hasan clarifies in his theoretical framework that members of the council, or whatever name applies, constitute jurists that reach to the rank of *ijtihad,* jurists who are not yet *mujtahidūn* and researchers in Islamic studies (Hasan 2003). However, in a practical sense, it is very challenging to ensure the involvement of a *mujtahid* in such committees. Nonetheless, the involvement of jurists who are knowledgeable in applying the principles of Islamic jurisprudence is essential. Collective *ijtihād* cannot be achieved without consultative approach. The involvement of other experts in various fields is vital, thus justifying the pragmatism of Islamic law (Hasan 2003; Kamali 1996; Auda 2010). The multidisciplinary committee contributes towards the concrete verdicts for Muslim communities especially in the complex issues such as biomedical ethics. Consultation between the *muftis* and medical scientists has become essential due to increasing scientific and technological advances (Padela 2007).

Both consultative and collective approaches are primary criteria for the purpose of this research in selecting institutional fatwa organisations, including those at national, regional and international levels. Table 1 shows the list of particular juristic councils that are chosen and observed via online and offline inputs. These councils are accessible in English, Arabic or Malay.

**Table 1:** Different levels of juristic councils

Level	Juristic councils
International	International Islamic Fiqh Academy Islamic Fiqh Academy Muslim World League
Regional	European Council for Fatwa and Research
National	Council of Senior Scholars Saudi Arabia General Presidency of Scholarly Research and Iftāʾ Saudi Arabia Dār al-Iftāʾ Egypt General Authority of Islamic Affairs and Endowment UAE Islamic Council for Fatwa Bayt al-Maqdis Islamic Fiqh Academy India Fatwa Committee of The National Council for Islamic Religious Affairs, Malaysia Islamic Religious Council of Singapore Governmental Mufti Department Brunei Religious Council of Indonesia

There are two juristic councils with similar names at the international level which are in themselves seen as connecting platforms for Muslim around the globe, namely the International Islamic Fiqh Academy (*al*-*Majmaʿal*-*Fiqh al*-*Islāmiy al*-*Duwaliy*) (1993) and Islamic Fiqh Academy Muslim World League (*al*-*Majmaʿ al*-*Fiqh al*-*Islāmiy Rābiṭah al*-*ʿĀlam al*-*Islāmiy*) (2004). At the European regional level, European Council for Fatwa and Research (2016) is a council that has been established in 1997. A number of juristic councils have also been established at the national level especially in Middle Eastern countries including Council of Senior Scholars Saudi Arabia (Hayʾah Kibār al-ʿUlamaʾ 2018), General Presidency of Scholarly Research and Ifta (n.d.), *Dār al-Iftāʾ al-Miṣriyyah* (2016), General Authority of Islamic Affairs and Endowment (United Arab Emirates 2016; The Islamic Council for Fatwa Bayt al-Maqdis 2012).

The Islamic Fiqh Academy India (2012) is one example in Asia. In southeast Asia, the juristic councils of Islamic Religious Council of Singapore (2016), and the Governmental Mufti Department Brunei Darussalam (2016) are the governmental bodies that regulate fatwa for their citizen. In Malaysia, the Fatwa Committee of the National Council of Islamic Religious Affair is a body comprised of 14 jurist consults from all states and selected professionals from various backgrounds. It is overseen by the federal government institution, Department of Islamic Development Malaysia (known as JAKIM) (Fatwa Management Division 2016). Lastly in Indonesia, Majelis Ulama Indonesia (2016), a non-governmental body consisting of 26 scholars from 26 provinces in Indonesia, has been established in 1975 to provide Islamic legal verdicts as one of their roles (Ichwan 2005). Although MUI is not a statutory body, anchored by law, it continues to be funded by the government and provides religious advice for government and communities (Ichwan 2005).

To analyse the aforementioned issues, the compilation of fatwas has been thematically classified into four categories, which are: (1) the connection of *khunūthah* and DSD, (2) gender assignment, (3) decision-making process and (4) surgical and hormonal treatments.

## Result

Table 2 provides status information on fatwas published by various juristic councils on the four issues on managing people with gender ambiguity. None of the juristic councils have explained the two spectrums of *khunthā* and DSDs in detail. The most probable reason is the fatwa decision which is usually based on the inquiry. Therefore, depending on the requests received, the deliberation may not be overarching. Out of thirteen councils observed, four of them, namely European Council for Fatwa and Research, Dār al-Iftāʾ Egypt, Islamic Religious Council of Singapore and Governmental Mufti Department Brunei, provide no answer to the aforementioned issues of DSDs. Nine of them provide a verdict on at least one of the four issues directly or indirectly as follows.

**Table 2:** Fatwas from different juristic councils over the four main issues of khunūthah and DSD

	Juristic councils	*Khunūthah* and DSDs	Gender assignment	Decision-making process and confidentiality	Surgical and hormonal treatment
1	International Islamic Fiqh Academy	–	–	Fatwa No. 79 (10/8) (1A)	–
2	Islamic Fiqh Academy Muslim World League	–	11th conference 1989 (2A)	–	11th conference 1989 (2A)
3	European Council for Research and Fatwa	–	–	–	–
4	Council of Senior Scholars Saudi Arabia	–	Fatwa No. 176/1992 (4A)	–	Fatwa No. 176/1992 (4A) Fatwa No. 119/1984 (4B)
5	General Presidency of Scholarly Research and Iftāʾ Saudi Arabia	–	Fatwa No. 21058/1999 (5A)	–	Fatwa No. 9085 (5B)
6	Dār al-Iftāʾ Egypt	–	–	–	–
7	General Authority of Islamic Affairs and Endowment, Kuwait	–	–	–	Fatwa No. 2571/2008 (7A)
8	Islamic Council for Fatwa Palestine	–	–	–	Fatwa No. 658/2012 (8A)
9	Islamic Fiqh Academy India	–	–	Included in fatwa No. 33 (8/1) (9A)	Included in fatwa No. 78 (18/3) (9B)
10	Fatwa Committee of The National Council of Islamic Religious Affairs, Malaysia	Included in 76th conference, 2006 (10A)	–	76th conference, 2006 (10A)	4th conference, 1982 (10B) 25th conference, 1989 (10C) 76th conference, 2006 (10A)
11	Islamic Religious Council of Singapore	–	–	–	–
12	Governmental Mufti Department Brunei	–	–	–	–
13	Religious Council of Indonesia	–	Fatwa no. 3/MUNAS-VII/MUI/2010 (13A)	Fatwa no. 3/MUNAS-VII/MUI/2010 (13A)	Fatwa no. MUNAS II/1980 (13B) Fatwa no. 3/MUNAS-VII/MUI/2010 (13A)

### Khunthā and DSDs

A clear relationship between a *khunthā* and DSDs is not addressed in any fatwa. None of them relate the discussion of *khunthā* with the current biomedical nomenclature except that which has been stated in the decision made by the Fatwa Committee of the National Council for Islamic Religious Affairs Malaysia (10A) (2010). The Committee states two types of DSDs regarding the permissibility of genital reconstruction without clearly mentioning whether those two types are recognised as *khunthā* or not. The medical nomenclature used in this verdict may be familiar to the physicians but obscure for laypersons.

### Gender Assignment

Members of the International Islamic Fiqh Academy and the Council of Senior Scholars of Saudi agree that all dominant signs that depict either the male or female are referred to in order to assign the gender for *khunthā ghayr mushkil* (determinate). Instead, if there is no dominant sign to confirm either the masculinity or femininity, such persons will remain as *khunthā mushkil* (indeterminate) as stated by the juristic councils of Saudi Arabia (n.d.), United Arab Emirates (2016) and Bayt al-Maqdis (2012) in fatwa 5A, 7A and 8A, respectively. In Indonesia, the fatwa (13A) does not provide the criteria of gender assignment but clarifies the assigned gender based on medical findings implies its Islamic legal ruling immediately even before the confirmation of the courts regarding the status of the reassigned sex (Majelis Ulama Indonesia 2011).

### Decision-Making Process

Despite the idea of having representatives of Islamic experts in managing patients with DSDs, juristic councils often do not make any suggestions whatsoever for the involvement of Muslim scholars in the medical multidisciplinary team of managing patients with DSDs. Most of the available fatwas do emphasise the importance of referring to physicians in order to ensure the appropriate signs of the dominant sex and the ensuing treatment. For instance, the juristic council of United Arab Emirates (2016) highlights the significance of a specialised medical team, as their decision will result in peculiar individual conduct and Islamic juridical rulings (7A).

Having a non-medical expert in a team would lead to the exchanging of information and thus involve issues of confidentiality, one of the core obligations of a doctor, as stated by the Islamic Fiqh Academy India (2012) in fatwa 9B. Though, based on fatwa of International Islamic Fiqh Academy (1993) (1A), an infringement of confidentiality is permissible if there is the need to curtail harm on individuals or the society. The Academy (1993) also recommends proper and in-depth procedures on how and to whom the information should be reported.

### Surgical and Hormonal Treatments

This aspect is the most discussed by juristic councils. In this sense, five related fatwas (2A, 4A, 8A, 10B and 13A) base their arguments on a reminder of the prohibition of altering Allah’s creation to ensure that the fatwa is only applicable for *khunthā*, since it is regarded as an impairment (Islamic Fiqh Academy Muslim World League 2004; Al-Fawzān 1424H; The Islamic Council for Fatwa Bayt al-Maqdis 2012; Department of Islamic Development Malaysia 2010; Majelis Ulama Indonesia 2011). Thus, reconstructive surgery is allowed to eliminate any defect, according to the Islamic Fiqh Academy India (2012) (9B). In Malaysia, there is progress in explaining the condition with regard to the treatment, from a plain statement (10B and 10C) to a more detailed explanation (10A) (Department of Islamic Development Malaysia 2010). A clearer guideline can be found in the Bayt al-Maqdis’ fatwa (2012) (8A), which states that there should be confirmation on (1) health risk condition; (2) surgery as the last resort of the treatment; (3) high probability of benefits by assigning the gender; (4) acceptance of the surgical treatment by the patient; and (5) availability of expert surgeons and their assistants.

Treatment is permissible through either a hormonal or surgical approach for *khunthā ghayr mushkil* (determinate) whenever the physical characteristics of maleness or femaleness are clear (2A, 4A, 7A, 8A, 10B) (Islamic Fiqh Academy Muslim World League 2004; Al-Fawzān 1424H; United Arab Emirates 2016; The Islamic Council for Fatwa Bayt al-Maqdis 2012; Department of Islamic Development Malaysia 2010). In contrast, surgical treatment is not allowed for *khunthā mushkil* whenever the signs of masculinity or femininity are vague so as to avoid any futile act as noted in fatwa 5B of Saudi Arabia, and marriage is prohibited for them (5B, 7A) (General Presidency of Scholarly Research and Ifta n.d., United Arab Emirates 2016). Unlike these latter fatwas, fatwas 10B and 10C in Malaysia note that *khunthā mushkil* who retain both the male and female genitalia are allowed to undergo surgical treatment, leaving the status of *khunthā ghayr mushkil* undefined (Department of Islamic Development Malaysia 2010).

Any surgical treatment cannot be conducted without the consent of the legally major and rational patient or the approval of the guardian for a minor patient as stipulated in fatwa 4B of Council of Senior Scholars Saudi Arabia (Al-Fawzān 1424H).

## Discussions

To find solutions for all issues from single juristic council seems insufficient. To understand contemporary views from Islamic perspective is to look at the responses globally. One of the issues remains obscure in terms of any juridical opinion is the elucidation of the connection between *khunthā* and DSDs. It can be argued that literal explanation of *khunthā* and DSDs should go beyond the role of the fatwa itself as a medium to expound such Islamic legal rulings for patients with DSDs. This is essential to filling the gap between the classical *fiqhi* understanding of *khunthā* and current medical findings of sex ambiguity.

In addition, contemporary medical nomenclature changes by the dynamism of the research and social context, and thus, it will affect the credibility of fatwa as the reference of majority Muslim communities in future. Therefore, I argue that any current fatwa that reflect new insights of biomedical findings can provide clear magnitude of explanation on this particular condition.

Before this, it is vital to align the justification of such rulings by one juristic council to another. As the aforementioned, there is difference between the term of *khunthā mushkil* alluded to by majority of juristic councils and the one provided by Fatwa Committee of the National Council of Religious Islamic Affairs Malaysia. While the majority of opinions prohibit surgical treatment for that particular type of *khunthā,* fatwa in Malaysia tends towards permissibility. Perhaps the difference is due to misunderstanding that *khunthā mushkil* is the only type that is associated with dual genitalia, as stated by Norliah Sajuri (2006), an officer at Department of Islamic Development Malaysia. Conversely, *mushkil* itself in Arabic refers to doubtful condition until there is evidence to resolve the complexity (ʿAbd al-Ḥamid 2008), no matter which form of genital ambiguity it is. Hence, a clear definition of the term in relation to the genotype and phenotype of a person is expected to be able to eliminate any confusion and misconception.

Fatwas on gender assignment have enlightened other experts seeking to understand and deliberate the issue from Islamic perspectives, as stated by Al-Jurayyan (2011a, b) a paediatrician in Saudi Arabia; Mohamed and Mohd Noor (2014a, b) senior lecturers in Malaysia; and Zainuddin and Mahdy (2016) paediatricians in Malaysia. They recognise *al*-*ʿalāmāt* or the signs of male or female as mentioned in the fatwas as evidence in determining gender. Al-Ḥaṭṭāb al-Ruʿīnī (1992) states that the method of seeking for critical assumptions is legally accepted in Islamic juridical thoughts. He bases his argument upon the Quranic exegesis on the incidents happened upon the Prophet Yūsuf where the *ʿalāmāt* suggested the actual situations (Al-Ḥaṭṭāb al-Ruʿīnī 1992). The context of the phenomenon is absolutely different, because the verses are explaining the accusation being made towards Prophet Yūsuf to seduce Zulaikha. Despite this incident, what is deducible from the verses 26–29 of *Sūrah* (Chapter) *Yūsuf* is the technique of deducing such rulings (*istinbāṭ al*-*ḥukm*) through comprehension of related indications.

Dessouky (2001) asked religious experts about the components of the word ‘signs’ in terms of whether it includes chromosomal sex, the gonadal sex, the phenotype, the appearance and function capability of external genitalia. To date, juristic councils provide no answer to that question, leaving them for the doctors to decide. Al-Barr, a medical consultant in King Fahd Centre for Medical Research, was in favour of this approach when he presented the paper in a conference of Islamic Fiqh Academy Muslim World League. He clarified that doctors have the ability to assign the gender based on the chromosomal and gonadal factors and afterwards religious scholars could deal with the patients in terms of juridical rulings based on the medical recommendations (Al-Sibāʿi and Al-Barr 1993). Al-Jurayyan (2011a, b) translate the word *ʿalāmāt* in the fatwa of Council of Senior Scholars Saudi Arabia as evidence and contextualise the word into medical diagnostics. According to Haneef and Abdul Majid (2015), experts of Islamic law in Malaysia, even seeking for assistance from medical experts to be useful, the jurist consults themselves should be acquainted with the cause of rulings or known as *taḥqīq al*-*manāṭ* in the principle of Islamic jurisprudence. In this case, the cause of rulings is related to biological development of the sexual anatomy.

It is clear that biological evidence is vital in seeking the ‘correct’ gender. Although it is not mentioned in the fatwas, the jurists opine that psychosexual orientation is another additional sign that can be taken into consideration when all other signs are not appropriate (Al-Suyūṭī 2001). In contrast, despite cultural or parental favour over specific gender, the medical findings and the guidelines from the Islamic perspective should not be ignored.

This issue is not unrelated with the decision-making process. It is quite odd for non-medical experts to make suggestions on the ‘correct’ gender or on the preferred treatment. But their involvement does contribute to ensuring the patients’ quality of health. Take for example a case of CAH patient as presented by Zainuddin and Mahdy (2016). It had been consulted by a religious authority in Malaysia. The consultation was fruitful for the patient to be a boy after long struggle with his biological conditions and refusal of being female Muslim. In the case of Malaysia, religious authorities can provide guidance on individual religious obligations. But what is more important is their approval pertaining to the changing of gender status in identification cards. This can curtail social life pressures such as attending school, finding a suitable job and even marriage.

The fatwas on gender assignment for *khunthā* ultimately provide clear support of surgical and hormonal treatment, despite lucid reminders that the change of sex for straight men and women as religious doctrinal crime due to changing what Allah has created. Dessouky (2001), a medical practitioner in Egypt, requested clear guidelines with regard to the legality of gonadectomy or excision of uterus in cases of females with Partial Androgen Insensitivity Syndrome (PAIS) and misassigned males with CAH especially in the late presentation of undiagnosed DSDs. Apparently, the verdict published by Fatwa Committee of the National Council of Islamic Religious Affairs Malaysia (Department of Islamic Development Malaysia 2010) manifests the permissibility of gonadectomy in the case of androgen insensitivity syndrome ‘for the prevention of malignancy’ (Zainuddin and Mahdy 2016).

Muslim jurists have welcomed the advancement of biomedical technology to bring about better chances to identify and rectify ambiguous external genitalia (Haneef and Abdul Majid 2015). The general guidelines as stipulated in the fatwa of The Islamic Council for Fatwa Bayt al-Maqdis (2012) are certainly accommodating the medical practitioners. Surgery is viewed as the last resort if hormonal treatment or other alternative is inappropriate. This fatwa might address Haneef and Abdul Majid’s (2015) qualm whether ‘is it justified in Islamic law to resort to the surgery in the first place?’ Their concern should be thoroughly contemplated because surgery is not only having potential health risks, but the procedures also involve in mutilation of human body parts, ravaging another’s private parts (which are privileged as private in Islam) and can cause mental and emotional harms (Haneef and Abdul Majid 2015).

Haneef and Abdul Majid (2015) urge Islamic jurists and jurist consults to go beyond the notion of *maṣlaḥah* in protecting life and family to legitimate the surgical treatment due to ethical issues such as unguaranteed true sex assignment, agony that the children have to face in which the surgery has been conducted while they are unable to give any consent, and manipulation of human genitalia if the outcome is uncertain.

## Concluding Remark

This article focuses on fatwas provided by the selected institutionalised jurist councils around the globe related to issues of sex ambiguity. While Muslim scholars have deliberated upon issues on sex ambiguity or *khunūthah*, its related rulings and gender assignment, biomedical advances have continued to explore numerous aspects of DSDs ranging from the terminology to diagnosis, gender assignment, treatments and other ethical spheres. Hence, the connections between them must be established to properly address the issues and the management of Muslim individuals affected by this condition.

While the institutions of fatwa develop their roles in various issues of biomedical debates, little has known on the management of DSDs. Based on the findings, the sporadic fatwas from different parts of the world delineate the issue of sex ambiguity and seem to be able to provide general guidelines for management of Muslim patients with DSDs. Three common aspects have been discussed including the methodology of gender assignment, the decision-making process and the surgical and hormonal treatments.

While several areas are not treated in much detail, the available verdicts at least open the way for further research from both the Islamic and medical perspectives. This includes the connection between limited scope of *khunthā* and the overarching term of DSDs. Guidelines of gender assignment and issues related to the decision-making process also require in-depth research as well.

These fatwas not only benefit people in a specific geographical boundary, but the accessibility through online and offline publications makes them available for people from other part of the globe. Certainly, fatwas link the past and the future, bridge the gap between Islamic and biomedical perspectives, put Islamic scholars and medical practitioners together and connect Muslims around the world.
